# Association of *SCAP* Gene Polymorphisms with Ambulatory Blood Pressure Parameters in College Students

**DOI:** 10.3390/genes17070825

**Published:** 2026-07-19

**Authors:** Yuan Zeng, Bin Mao, Jian Zhang, Sha Xia, Zhe Wu, Shang Li, Xiuqin Hong, Yide Yang

**Affiliations:** 1 Changsha Municipal Center for Disease Control and Prevention, Changsha 410004, China; 2 Key Laboratory of Molecular Epidemiology of Hunan Province, School of Public Health, Hunan Normal University, Changsha 410081, China; 3 Hunan Provincial People’s Hospital Affiliated to Hunan Normal University, Changsha 410021, China

**Keywords:** ambulatory blood pressure, *SCAP* gene, college students, interaction effect

## Abstract

**Background/Objectives**: Abnormal blood pressure (BP) in youth is strongly linked to genetic predisposition, particularly involving lipid metabolism genes. However, little is known about whether such polymorphisms affect ambulatory BP (ABP) parameters and whether other factors modify the associations in youth populations. **Methods**: A total of 510 medical students from a university in Changsha were included. Multivariable linear regression and logistic regression models were used to analyze the association between sterol regulatory element-binding protein cleavage-activating protein (*SCAP*) gene polymorphisms and ABP parameters among youth. **Results**: After adjusting for age, sex, BMI, ethnicity, monthly household income per capita, salt intake habits, fruit and vegetable intake frequency, smoking, drinking, history of hypertension and waist circumference, carriers of the A allele of rs76558868 had higher daytime systolic blood pressure (SBP) than G allele carriers (*β* = 1.55, SE = 0.77, *p* = 0.046, corrected *p* = 0.092). A significant interaction between rs12487736 and sex on 24 h SBP (*p_interaction_* = 0.023) and daytime SBP (*p_interaction_* = 0.048) levels was found. In males, the CC genotype carriers had elevated 24 h SBP (*β* = 3.53, SE = 1.36, *p* = 0.010, corrected *p* = 0.020) and daytime SBP (*β* = 3.18, SE = 1.53, *p* = 0.040, corrected *p* = 0.080) levels compared to TC/TT genotype carriers, whereas no significant association was found in females. In addition, we also noted an interaction between rs76558868 and sex on 24 h SBP (*p_interaction_* = 0.016), nighttime SBP (*p_interaction_* = 0.027) and nighttime diastolic blood pressure (DBP) (*p_interaction_* = 0.025) levels; the A allele carriers had significantly higher 24 h SBP (*β* = 2.02, SE = 0.84, *p* = 0.018, corrected *p* = 0.036), nighttime SBP (*β* = 2.64, SE = 0.98, *p* = 0.008, corrected *p* = 0.016) and DBP levels (*β* = 1.65, SE = 0.63, *p* = 0.009, corrected *p* = 0.018) compared to G allele carriers among males, but not in females. **Conclusions**: An interaction between *SCAP* gene polymorphisms and sex was observed for ABP parameters among college students. These findings provide insights for future targeted early hypertension prevention and personalized health management strategies.

## 1. Introduction

Hypertension is one of the most important risk factors for cardiovascular disease and premature death worldwide, and high systolic blood pressure (SBP) was one of the leading risk factors globally in 2023 (8.4% of total disability-adjusted life-years) [1,2]. There has been a rapid increase in the prevalence of global hypertension, especially among young individuals [3]. Evidence from a meta-analysis indicated that the prevalence of hypertension nearly doubled among children and adolescents aged 19 years or younger from 2000 to 2020 (boys: 3.40% to 6.53%; girls: 3.02% to 5.82%), and the prevalence of hypertension measured by the in-office method and the combined in-office and out-of-office assessments was 4.28% and 6.67%, respectively [4]. Ambulatory blood pressure (ABP) can present the true fluctuation of an individual’s blood pressure (BP) more comprehensively through 24 h monitoring (such as the dipping phenomenon), which also reduces the stress reaction that contributes to white-coat hypertension and is more sensitive for the identification of masked hypertension, compared with in-office BP measurements [5]. It suggests that ABP monitoring can facilitate more valuable information for the potential cardiovascular risk assessment and earlier intervention in the management of college students.

Environmental exposure and lifestyle factors are well-documented contributors to hypertension, and genetic susceptibility is also of vital importance to the development of hypertension [6,7]. Accordingly, the efficacy of drugs for treating hypertension in the population is also closely interrelated with genes [8]. Sterol regulatory element-binding protein cleavage-activating protein (SCAP) is a protein closely related to the regulation of lipid metabolism. It is a key regulatory factor in the SREBP pathway, participating in regulating the cholesterol and lipid synthesis pathways [9,10]. It binds to the sterol regulatory element-binding protein (SREBP) via its COOH-terminal domain to form the SCAP-SREBP complex [11]. At high cholesterol concentrations, SCAP undergoes conformational changes and binds to the insulin-induced gene protein (INSIG), retaining the INSIG/SCAP/SREBP complex in the endoplasmic reticulum and suppressing cholesterol synthesis. At low cholesterol concentrations, the complex dissociates from INSIG and translocates to the Golgi apparatus, where proteolytic cleavage releases transcription factors that activate target genes involved in cholesterol and lipid metabolism, thereby enhancing lipid synthesis [12]. Growing evidence suggests that the circadian rhythm regulatory system mediates lipid metabolism and maintains lipid homeostasis [13,14]. ABP captures the full 24 h circadian profile of blood pressure, which is particularly relevant, given SCAP’s potential role in circadian-regulated lipid metabolism [15,16]. Indeed, a recent study observed that SCAP was required for Reverb-hDKO-induced diurnal rhythmic remodeling and epigenomic reprogramming in liver macrophages [16]. However, no published study has specifically investigated the association between *SCAP* gene polymorphisms and 24 h ABP parameters. All prior evidence linking *SCAP* gene polymorphisms to blood pressure has been derived from office blood pressure measurements in pediatric populations [17,18]. Therefore, extending the investigation of SCAP polymorphisms to 24 h ABP parameters in young adult populations addresses this knowledge gap and may reveal circadian-specific associations that are not detectable using conventional office blood pressure measurements.

A study has reported that obese patients had increased ABP parameters and abnormal circadian blood pressure rhythms, and the prevalence of non-dipping blood pressure status was also higher [19]. Other research has found that the day–night blood pressure patterns of people with different weight statuses are also inconsistent [20]. Our previous research has also reported that rs12490383 and rs12487736 polymorphisms were significantly associated with high SBP among Chinese children and adolescents who were overweight or obese [17]. A case-control study demonstrated a significant interaction between the rs12487736 and the intake of high-calorie foods, such as French fries, cakes, and cookies, suggesting that lifestyle behaviors and the gene polymorphism may jointly affect BP levels in children and adolescents [18]. Nevertheless, Zhou et al. found that severe obesity was associated with a higher prevalence of non-BP dipping patterns in girls than in boys, which suggests that the relationship between the severity of obesity and BP dipping status might be sex-specific [21].

This study aimed to explore the association between the *SCAP* gene polymorphisms and the ABP parameters [24 h SBP, 24 h diastolic blood pressure (DBP), daytime SBP, daytime DBP, nighttime SBP, nighttime DBP, abnormal 24 h BP, abnormal daytime BP, and abnormal nighttime BP] in college students. Additionally, whether *SCAP* gene polymorphisms could interact with weight status and sex to affect ABP parameters has not been demonstrated before. Thus, we also explored the interactions between the polymorphisms of the *SCAP* gene and sex, as well as with weight status.

## 2. Subjects and Methods

### 2.1. Subject

A total of 514 medical students from a university in Changsha participated in on-site investigations, physical examinations, ABP monitoring, and genotyping tests from September 2022 to June 2024.

Inclusion criteria: (1) participation in the university pre-enrollment medical examination; (2) having relevant information on demographic characteristics, lifestyle, history of hypertension, and physical examination indicators; (3) collection of a peripheral blood sample; (4) availability of 24 h ambulatory blood pressure monitoring data. Exclusion criteria: (1) missing questionnaire information or physical examination data; (2) no peripheral blood sample; (3) no 24 h ambulatory blood pressure monitoring data; (4) current use of antihypertensive medication; and (5) presence of severe cardiovascular disease or major organ diseases such as liver or kidney dysfunction.

Four participants were excluded for invalid questionnaire data (n = 1), missing 24 h ABP data (n = 1), and missing physical examination indicators (n = 2). Therefore, a total of 510 participants were included in the present study. The cross-sectional study was approved by the Medical Research Ethics Review Committee (2019-88, 9 March 2019) of the Hunan Normal University under the guidelines of the Declaration of Helsinki.

### 2.2. Sample Size Calculation

Sample size was calculated using Quanto 1.2.4 software for a genetic association study. Assuming a prevalence of abnormal 24 h BP of 6.67% [4], an odds ratio (*OR*) of 1.62 [22], a minimum allele frequency (MAF) between 0.16 and 0.41, and a statistical power of 80%, the required sample size was estimated to be 446 participants.

### 2.3. Measurement

Height (cm) was measured using a sit-down height gauge, and weight (kg) was determined using a bioelectrical impedance body composition analyzer [TANITA MC-780MA, Japan (TANITA (SHANGHAI) TRADING Co., Ltd., Shanghai, China)]. Height and weight are used to calculate BMI (kg/m^2^). The waist circumference (WC, cm) is measured using a tape measure. For participants aged 18 and above, overweight/obesity is defined as a BMI ≥ 24 kg/m^2^ [23]. For participants under 18 years of age, overweight and obesity were classified according to the screening standards among children and adolescents published by the National Health Commission of China (standard number WS/T 586-2018) [24]. Approximately 3 mL of venous blood (stored at −20 °C) was collected from each participant. Blood draws were performed in the early morning (approximately 7:00–8:30 AM) after an overnight fast of at least 8 h. The participants self-reported their age, gender, ethnicity, salt consumption habits, average monthly household income, fruit and vegetable intake in the past week, smoking and drinking status, and history of hypertension [at least one first-degree relative (parent or sibling) had hypertension] [25].

### 2.4. 24 h ABP Monitoring

Participants’ 24 h ABP monitoring was performed using the TM-2430 ambulatory blood pressure monitor [A&D, Japan (A&D Technology Trading (Shanghai) Co., Ltd., Shanghai, China)]. The cuff of the monitor was worn on the non-dominant arm of the participants. BP was measured every 20 min during the day (from 7 a.m. to 11 p.m.) and every 30 min during the night (from 11 p.m. to 7 a.m.). Moreover, the average SBP and DBP during the day and night were determined from valid measurement values during all waking hours and sleep time (recorded in the sleep diary). All participants completed the complete 24 h ABP monitor record as defined by the European Society of Hypertension (with more than or equal to 70% of the planned readings and at least 20 daytime and 7 nighttime readings) [26]. The ABP monitoring indicators included 24 h SBP, 24 h DBP, daytime SBP, daytime DBP, nighttime SBP, nighttime DBP, abnormal 24 h BP, abnormal daytime BP, and abnormal nighttime BP. Abnormal 24 h BP was defined as 24 h SBP ≥ 130 mm Hg and/or 24 h DBP ≥ 80 mm Hg; abnormal daytime BP was daytime SBP ≥ 135 mm Hg and/or daytime DBP ≥ 85 mm Hg; abnormal nighttime BP was nighttime SBP ≥ 120 mm Hg and/or nighttime DBP ≥ 70 mm Hg [27].

### 2.5. Genetic Polymorphisms Selection and Genotyping

We selected the positive results or the sites with more studies from the previously published literature on the association between the *SCAP* genes and cardiovascular metabolic risk factors. Additionally, it included tagging a single-nucleotide polymorphism (Tag SNP) for analysis in the *SCAP* gene. The Tag SNP refers to a specific type of single nucleotide polymorphism (SNP) that is representative in the genome. The sites that met the Hardy–Weinberg equilibrium test and had an *r*^2^ < 0.80 for linkage disequilibrium analysis were included in the study. Finally, two SNPs from the *SCAP* gene [rs12487736 [18] and rs76558868(Tag SNP)] were selected.

DNA samples were extracted from venous blood samples using the salt-out method and under identical experimental conditions (such as temperature and time). Matrix-Assisted Laser Desorption/Ionization Time of Flight Mass Spectrometry (MALDI-TOF MS, Agena) technology was used for the genotyping. Replicate samples were set up during the genotyping process, and the genotyping technicians were blinded to the identity of the duplicate samples. The genotyping results of the replicate samples were consistent with those of the original samples. Additionally, the call rate for all polymorphisms was 100% (Supplementary Table S1).

### 2.6. Statistical Analysis

The general characteristics, lifestyle, history of hypertension, and 24 h ABP monitoring indicators of the subjects were described. Continuous variables and categorical variables were described using mean ± SD and n (%), and *t*-tests or rank-sum tests were used to analyze the differences in continuous variables; chi-square tests were used to compare the differences between groups of categorical variables. The Hardy–Weinberg equilibrium test was used to assess the genotype of the normal ABP group of college students (*p*_-HWE_ > 0.05 indicated genetic equilibrium in the population). The linkage disequilibrium relationship was analyzed using Haploview 4.2 software (*r*^2^ < 0.8 determined linkage equilibrium). The difference in a certain allele between races was evaluated using the F-statistics formula (*F_ST_* = (P_1_ − P_2_)^2^/[(P_1_ + P_2_) × (2 − (P_1_ + P_2_))]; P_1_ and P_2_ represent the MAF of the gene in the European population and the study population, respectively) [28]. The *F_ST_* values were divided into four categories: small racial difference (0 ≤ *F_ST_* < 0.05), moderate (0.05 ≤ *F_ST_* < 0.1), large (0.15 ≤ *F_ST_* < 0.25), and very large (*F_ST_* ≥ 0.25) [29]. Multivariable linear regression was used to analyze the association of genetic polymorphisms with the 24 h ABP parameters (24 h SBP, 24 h DBP, daytime SBP, daytime DBP, nighttime SBP, and nighttime DBP) under three genetic models (additive, dominant, and recessive). At the same time, the binary logistic regression method was used to evaluate the association between genetic polymorphisms and abnormal BP phenotypes (abnormal 24 h/daytime/nighttime BP). The genetic model was defined by *SCAP*/rs76558868, and as an example, where A was the effect allele, the additive model was GG = 0, AG = 1, and AA = 2; the dominant model was GG = 0, and AG/AA = 1; the recessive model was GG/AG = 0, and AA = 1. According to the AIC criterion, the optimal genetic model of the *SCAP* genes was selected, and the interaction terms of genetic variation and gender and weight status (normal, overweight/obesity) were included in the general linear model to evaluate their influence on the ABP levels, and a stratified analysis by sex and weight status was conducted. Considering that *SCAP* in the present study selected 2 SNPs, we adjusted multiple testing for the Bonferroni correction. Statistical analysis was performed using IBM SPSS Statistics 27.0 software. The significance level was α = 0.050 (two-sided).

## 3. Results

### 3.1. General Characteristics

The study included a total of 510 students; 17 participants (3.3%) were categorized as abnormal 24 h BP, 16 (3.1%) with abnormal daytime BP, and 20 (3.98%) with abnormal nighttime BP. The characteristics of the individuals are shown in Table 1. The mean age was 18.29 ± 0.77 years, with 64.7% of females in the total sample. Females were more likely to consume fruit ≥ 1 per week, never smoke, never drink, have a normal weight, have a low SBP (24 h, daytime and nighttime SBP), DBP (24 h, daytime and nighttime DBP) and waist circumference (WC) levels, and have a lower prevalence of abnormal BP (abnormal 24 h, daytime and nighttime BP) compared to males. The ancestral differences in *SCAP* (rs12487736, rs76558868) gene polymorphisms between the European population and this study population are all small (all *F_ST_* < 0.05), as shown in Supplementary Table S1.

### 3.2. Associations Between SNP with ABP Parameters

According to the AIC criterion, the recessive genetic model showed the lowest AIC value for rs12487736 and the additive genetic model for rs76558868 (Supplementary Table S2).

In the recessive genetic model, using sex, age and BMI as covariates, a significant association between rs12487736 and nighttime SBP levels was observed (*β* = 1.75, SE = 0.87, *p* = 0.046), but after further adjustment of other covariates (ethnicity, monthly household income per capita, salt intake habits, fruit and vegetable intake frequency, smoking, drinking, history of hypertension and WC), no significant association was found (*β* = 1.55, SE = 0.87, *p* = 0.075). Moreover, rs76558868 was associated with daytime SBP levels, and individuals with A allele carriers had higher daytime SBP levels than G allele carriers (*β* = 1.55, SE = 0.77, *p* = 0.046) in an additive genetic model with full adjustment (Table 2). However, rs76558868 was not significantly associated with daytime SBP levels after correction for multiple comparisons (corrected *p* = 0.092).

No significant associations between the SNP polymorphism in *SCAP* and abnormal 24 h BP/daytime BP/nighttime BP levels were found (Supplementary Table S3).

### 3.3. Interaction Between SNP and Sex on ABP Levels

Significant interaction between *SCAP* gene polymorphism and sex was identified. With adjustment of age, weight status, monthly household income per capita, salt intake habits, fruit and vegetable intake frequency, smoking, drinking, history of hypertension and WC, the interaction between *SCAP* gene polymorphisms and sex on the ABP levels was further analyzed (Table 3).

**Figure 1:**
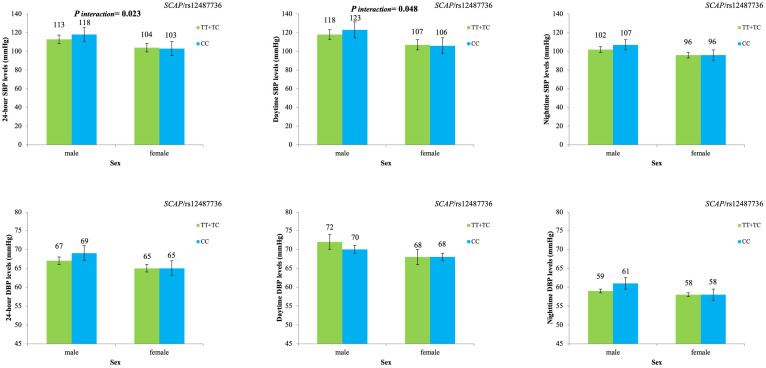
Adjusted means and standard errors of ambulatory blood pressure levels (24 h, daytime and nighttime blood pressure levels) stratified by *SCAP*/rs12487736 polymorphism and sex. Adjusted mean and standard errors were estimated under a general linear regression model that adjusted for age, body mass index group, ethnicity, monthly household income per capita, salt intake habits, fruit intake frequency, vegetable intake frequency, smoking, drinking, history of hypertension and waist circumference.

A significant interaction between rs12487736 and sex on 24 h SBP (*p_interaction_* = 0.023) and daytime SBP (*p_interaction_* = 0.048) levels was observed. The CC genotype carriers had significantly higher 24 h SBP (*β* = 3.53, SE = 1.36, *p* = 0.010, corrected *p* = 0.020) and daytime SBP (*β* = 3.18, SE = 1.53, *p* = 0.040, corrected *p* = 0.080) levels than TC/TT genotype carriers in males, but no significant association was found in females. Nevertheless, the association between rs12487736 and male daytime SBP levels was not significant (*β* = 3.18, SE = 1.53, *p* = 0.040, corrected *p* = 0.080) after multiple comparison corrections. The adjusted ABP levels stratified by different rs12487736 genotypes and sex are shown in Figure 1.

**Figure 2:**
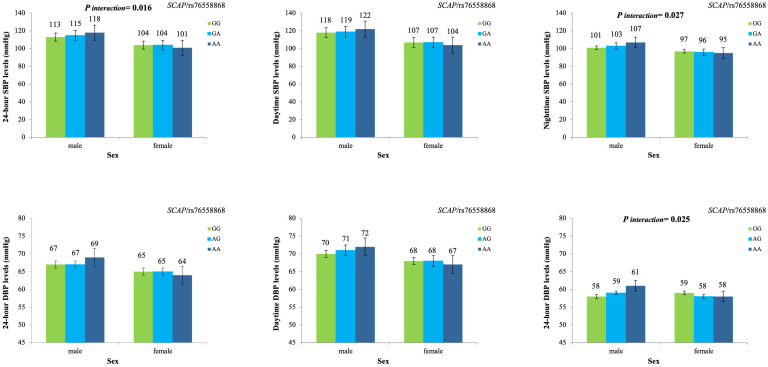
Adjusted means and standard errors of ambulatory blood pressure level (24 h, daytime and nighttime blood pressure levels) stratified by *SCAP*/rs76558868 polymorphism and sex. Adjusted mean and standard errors were estimated under a general linear regression model that adjusted for age, body mass index group, ethnicity, monthly household income per capita, salt intake habits, fruit intake frequency, vegetable intake frequency, smoking, drinking, history of hypertension and waist circumference.

Notably, there is an interaction between rs76558868 and sex on 24 h SBP (*p_interaction_* = 0.016), nighttime SBP (*p_interaction_* = 0.027) and nighttime DBP (*p_interaction_* = 0.025) levels. In males, the A allele was significantly associated with elevated 24 h SBP (*β* = 2.02, SE = 0.84, *p* = 0.018, corrected *p* = 0.036), nighttime SBP (*β* = 2.64, SE = 0.98, *p* = 0.008, corrected *p* = 0.016) and nighttime DBP levels (*β* = 1.65, SE = 0.63, *p* = 0.009, corrected *p* = 0.018) compared with G allele, whereas no significant association was observed in females. These associations remained significant after multiple comparisons corrections (all corrected *p* < 0.050). The adjusted ABP levels stratified by different rs76558868 genotypes and sex are demonstrated in Figure 2.

### 3.4. Interaction Between SNP and Weight Status on ABP Levels

With adjustment of age, gender, monthly household income per capita, salt intake habits, fruit and vegetable intake frequency, smoking, drinking, history of hypertension and waist circumference, the interaction between *SCAP* gene polymorphisms and weight status on the ABP levels of college students is presented in Supplementary Table S4.

No significant interactions were detected for the *SCAP* gene polymorphisms and weight status with ABP levels.

## 4. Discussion

In the present study, we examined an association between the polymorphisms of *SCAP* genes and the ABP phenotypes of Chinese college students and also identified the interaction between these polymorphisms with sex and weight status on the ABP levels (including 24 h SBP, 24 h DBP, daytime SBP, daytime DBP, nighttime SBP and nighttime DBP) among college students. Meanwhile, the results showed that rs76558868 was positively correlated with the daytime SBP levels, although this association did not survive after correction for multiple comparisons. Additionally, a significant sex-specific interaction was found for rs12487736 (24 h SBP and daytime SBP) and rs76558868 (24 h SBP, nighttime SBP and nighttime DBP) on ABP levels.

To the best of our knowledge, no study has specifically explored the relationship between rs76558868 and ABP levels. Our results suggest that rs76558868 was associated with daytime SBP levels in additive genetic models. However, the relationship between rs76558868 and daytime SBP levels should be interpreted with caution, as no significant association was found after correction for multiple comparisons, requiring further investigation in larger, independent cohorts. Our findings provide a new perspective for understanding the early genetic mechanisms of hypertension. Nevertheless, no association was observed between rs12487736 and the level of ABP among college students, but a previous study conducted on younger children (9–11 years) reported that rs12487736 has a positive correlation with office SBP and DBP levels [18]. The discrepancy may be attributable to several factors. First, the use of office BP in the previous study versus ABP in our study may have contributed to the different findings, as office BP and ABP capture distinct aspects of BP regulation. A recent network meta-analysis demonstrated that nighttime ABP is, on average, 18.14 mmHg lower than office SBP, and 24 h ABP is 8.63 mmHg lower, with discrepancies more pronounced at elevated BP levels [30]. These substantial differences highlight that office BP and ABP are not interchangeable measures and may yield different genetic association signals. Second, the developmental stage of the study population differs substantially; the earlier study included prepubertal children, whereas our participants were young adults who had completed pubertal growth. Blood pressure regulation and the influence of genetic variants may change across these developmental stages [31]. The current results emphasize the value of longitudinal tracking of genetic effects at different stages of life. For the same gene, its influence may dynamically change with age, environment, and developmental stage.

Interestingly, a significant interacting effect between gender and rs12487736 on 24 h and daytime SBP levels was identified. Specifically, in males, CC genotype carriers had higher levels of 24 h SBP and daytime SBP compared to TT/TC genotype carriers, which was not detected in females. Meanwhile, we also demonstrated interaction between rs76558868 and sex on the 24 h SBP, nighttime SBP and DBP levels. Compared with G allele carriers, the A allele carriers were associated with an increase in the 24 h SBP and nighttime SBP and DBP levels in males, while no association was observed in females. ABP follows a 24 h circadian rhythm that is closely intertwined with lipid metabolism. SCAP, as a master regulator of cholesterol homeostasis, may serve as a molecular link between these two circadian-regulated systems [15]. Recent evidence indicates that SCAP was required for Reverb-hDKO-induced diurnal rhythmic remodeling and epigenomic reprogramming in liver macrophages [16]. Thus, genetic variation in *SCAP* may influence blood pressure, at least in part, by modulating the amplitude or phase of circadian lipid rhythms. However, these assumptions still need to be further verified. Several mechanisms may potentially explain the sex-specific associations. First, androgens may amplify the effect of *SCAP* gene variations on BP, which directly regulates SCAP expression and overactivates the SREBP pathway, ultimately leading to abnormal lipid accumulation [32,33]. Second, androgens activate the renin–angiotensin–aldosterone system (RAAS) by enhancing renin release and angiotensin II generation, which in turn promotes vasoconstriction and sodium–water retention, ultimately increasing blood volume and peripheral vascular resistance [34]. This androgen-mediated pathway may potentiate the effects of *SCAP* genetic variation on blood pressure. Finally, androgens upregulate pro-inflammatory cytokines, including IL-6, TNF-α, and IL-1β, which contribute to endothelial dysfunction and vascular stiffening—key processes in hypertension development [35,36].

The variation in the *SCAP* gene may further activate the inflammatory pathway by enhancing lipid metabolism disorders, thereby causing abnormal blood pressure. In contrast, estrogen inhibits the SREBP pathway and subsequently reduces lipid synthesis [37] and induces eNOS expression to improve vascular dilation [38], which might explain the lack of a link between *SCAP* gene polymorphism and ABP levels among females. However, this interpretation remains speculative and requires validation in dedicated mechanistic studies.

A recent 18-year longitudinal study demonstrated that BMI significantly modifies genetic susceptibility to high SBP in adolescents and young adults. In 714 participants followed from ages 12 to 30, the association between a genetic risk score (GRS) and SBP levels increased monotonically with BMI values between 22 kg/m^2^ and 35 kg/m^2^ [39]. While we detected no significant interaction between *SCAP* gene polymorphisms and weight status, the Riglea et al. study [39] suggests that such interactions may require larger sample sizes or more extreme BMI ranges to achieve statistical power. Notably, the association in that study intensified primarily in the BMI 22–35 kg/m^2^ range—a range that may not be fully represented in our college students with generally lower BMI distributions. Beyond BMI, sleep duration has emerged as another critical environmental modifier of genetic susceptibility to blood pressure. A genome-wide gene-sleep duration interaction study in 811,405 individuals across five population groups discovered 22 novel gene-sleep duration interaction loci for blood pressure, and several of our loci are specific to a particular population background or sex [40]. Due to the absence of sleep duration and sleep quality data in this study, we were unable to explore the potential modifying effects of sleep phenotypes on the association between SCAP gene polymorphisms and ABP parameters. Future studies can further determine this interaction effect.

The major strengths of our study were that we concentrated on young college students and, by analyzing the abnormal ABP, highlighted the benefits for early risk identification and prevention of hypertension. Then, to the best of our knowledge, this is the first attempt to investigate the relationship between *SCAP* gene polymorphism and the ABP phenotypes, which provides new evidence for the genetic mechanism in blood pressure regulation.

As well, we acknowledge that the present study has several potential limitations. In the first place, the cross-sectional design precludes causal inferences regarding the relationship between *SCAP* polymorphisms and blood pressure phenotypes. Longitudinal studies are needed to establish temporal relationships and track BP trajectories. In the second place, the participants in the study are mainly composed of Chinese college students. Whether the observed relationship could be applied to other age groups and ethnic groups warrants further investigation. Third, although key covariates such as sex, diet, body weight status and antihypertensive medication use (none of the participants reported taking such medications) have been considered, other potential confounders that may affect ABP [such as sleep quality, other concurrent medications (lipid-lowering drugs or antidiabetic agents)] have not been investigated; residual confounding factors are inevitable. Fourth, while we have identified sex-specific associations between *SCAP* polymorphisms and circadian BP phenotypes, the exact functional mechanisms remain unknown. We did not perform functional research. Further functional studies are needed to elucidate the biological pathways underlying our observed associations. Ultimately, the relatively modest sample size (N = 510) also limited our ability to detect rare variants. In addition, the logistic regression analyses for abnormal BP phenotypes were likely underpowered because the number of abnormal BP cases is very small. Therefore, the negative findings regarding the abnormal BP phenotype should be interpreted with caution, and future studies with larger independent cohorts are warranted to validate these exploratory results.

## 5. Conclusions

In conclusion, we observed that the *SCAP* gene polymorphisms showed a sex-specific association with ABP parameters, and that the association is only significant in males. Our findings highlight that early risk assessment and intervention for hypertension are vital in young students. No significant interaction between *SCAP* gene polymorphisms and weight status was observed. These findings suggest a potential role for *SCAP* gene polymorphisms in circadian blood pressure regulation that may be modified by sex. Given the modest sample size and the exploratory nature of our analyses, these results should be interpreted cautiously and require validation in independent cohorts.

## Figures and Tables

**Table 1:** The characteristics of the population.

Variables	Total (n = 510)	Male (n = 180)	Female (n = 330)	*p*
Age (years)	18.29 ± 0.77	18.34 ± 0.79	18.26 ± 0.76	0.394
Ethnicity, n (%)				0.023
Han Chinese	455(89.2)	153(85.0)	302(91.5)	
non-Han Chinese	55(10.8)	27(15.0)	28(8.5)	
Monthly household income per capita, n (%)				0.044
<3000 CNY	111(21.8)	28(15.6)	83(25.2)	
3000–4999 CNY	122(23.9)	41(22.8)	81(24.5)	
≥5000 CNY	122(23.9)	47(26.1)	75(22.7)	
Unknown	155(30.4)	64(35.6)	91(27.6)	
Salt intake, n (%)				0.204
Salty	100(19.6)	29(16.1)	71(21.5)	
Moderate	346(67.9)	127(70.6)	219(66.4)	
Light	47(9.2)	15(8.3)	32(9.7)	
Unknown	17(3.3)	9(5.0)	8(2.4)	
Fruit intake frequency, n (%)				0.011
<1 time per week	405(79.4)	154(85.6)	251(76.1)	
≥1 time per week	105(20.6)	24(14.4)	79(23.9)	
Vegetable intake frequency, n (%)				0.169
<1 time per week	170(33.3)	53(29.4)	117(35.5)	
≥1 time per week	340(66.7)	127(70.6)	213(64.5)	
Smoking, n (%)				0.005
Yes	5(1.0)	5(2.8)	0(0.0)	
Never	505(99.0)	175(97.2)	330(100.0)	
Alcohol consumption, n (%)				<0.001
Yes	45(8.8)	31(17.2)	14(4.2)	
Never	465(91.2)	149(82.8)	316(95.8)	
History of hypertension, n (%)				0.483
Yes	105(20.6)	34(18.9)	71(21.5)	
No	405(79.4)	146(81.1)	259(78.5)	
Waist circumference (cm)	72.02 ± 8.26	75.53 ± 9.47	70.10(6.81)	<0.001
BMI (kg/m^2^)	21.53 ± 3.39	22.09 ± 3.73	21.22 ± 3.15	0.006
Weight status, n(%)				0.004
Overweight/obesity	111(21.8)	52(28.9)	59(17.9)	
Normal	399(78.2)	128(71.1)	271(82.1)	
24 h SBP (mmHg)	107.51 ± 10.11	114.84 ± 9.23	103.52(8.15)	<0.001
24 h DBP (mmHg)	66.03 ± 4.82	67.52 ± 4.97	65.22 ± 4.53	<0.001
Daytime SBP (mmHg)	111.10 ± 11.30	119.35 ± 10.31	106.61 ± 9.07	<0.001
Daytime DBP (mmHg)	68.97 ± 5.38	70.83 ± 5.52	67.95 ± 5.02	<0.001
Nighttime SBP (mmHg)	98.80 ± 9.72	103.47 ± 10.08	96.25 ± 8.50	<0.001
Nighttime DBP (mmHg)	58.75 ± 5.65	59.40 ± 6.12	58.40 ± 5.35	0.072
Abnormal 24 h BP, n (%)				<0.001
Yes	17(3.3)	15(8.3)	2(0.6)	
No	493(96.7)	165(91.7)	328(99.4)	
Abnormal daytime BP, n (%)				<0.001
Yes	16(3.1)	13(7.2)	3(0.9)	
No	494(96.9)	167(92.8)	327(99.1)	
Abnormal nighttime BP, n (%)				0.001
Yes	20(3.9)	14(7.8)	6(1.8)	
No	490(96.1)	166(92.2)	324(98.2)	

BMI: body mass index; SBP: systolic blood pressure; DBP: diastolic blood pressure; BP: blood pressure.

**Table 2:** The association between *SCAP* gene polymorphisms and ABP level.

Genes/SNPs	ABP Parameters	Model	N	SBP				DBP			
				*β*	SE	*p*	*p* ^a^	*β*	SE	*p*	*p* ^a^
*SCAP*/rs12487736 (Recessive genetic model)	24 h BP	1	510	1.29	0.80	0.107	0.214	0.52	0.45	0.253	0.506
		2	510	1.03	0.78	0.187	0.374	0.41	0.45	0.364	0.728
	daytime BP	1	510	1.01	0.89	0.256	0.512	0.29	0.50	0.563	1.000
		2	510	0.73	0.87	0.401	0.802	0.20	0.50	0.699	1.000
	nighttime BP	1	510	1.75	0.87	0.046	0.092	0.84	0.55	0.127	0.254
		2	510	1.55	0.87	0.075	0.150	0.73	0.56	0.191	0.382
*SCAP*/rs76558868 (Additive genetic model)	24 h BP	1	510	0.23	0.53	0.666	1.000	0.16	0.30	0.605	1.000
		2	510	0.22	0.51	0.674	1.000	0.16	0.30	0.597	1.000
	daytime BP	1	510	1.47	0.79	0.062	0.124	−0.79	0.45	0.077	0.154
		2	510	1.55	0.77	0.046	0.092	−0.75	0.45	0.091	0.182
	nighttime BP	1	510	0.55	0.58	0.344	0.688	0.23	0.37	0.523	1.000
		2	510	0.54	0.57	0.347	0.694	0.22	0.37	0.539	1.000

*SCAP*: sterol regulatory element-binding protein cleavage-activating protein, ABP: ambulatory blood pressure, SNPs: single nucleotide polymorphism, BP: blood pressure, SBP: systolic blood pressure, DBP: diastolic blood pressure, SE: standard error. Model 1 adjusted for sex, age and BMI; Model 2 further adjusted for ethnicity, monthly household income per capita, salt intake habits, fruit intake frequency, vegetable intake frequency, smoking, drinking, history of hypertension and waist circumference. ^a^: The *p*-values of adjusted multiple testing for Bonferroni correction.

**Table 3:** The interaction between *SCAP* gene polymorphisms and gender on ABP levels.

Genes	SNPs	Sex	Genotype	N	SBP					DBP				
					*β*	SE	*p*	*p* ^a^	*p_interaction_*	*β*	SE	*p*	*p* ^a^	*p_interaction_*
		**24 h ABP levels**												
*SCAP*	rs12487736	male	TT + TC/CC	125/55	3.53	1.36	0.010	0.020	0.023	1.25	0.81	0.124	0.248	0.225
		female		240/90	0.44	0.96	0.646	1.000		−0.08	0.55	0.880	1.000	
	rs76558868	male	GG/AG/AA	60/82/38	2.02	0.84	0.018	0.036	0.016	1.04	0.50	0.037	0.074	0.085
		female		120/169/41	0.96	0.64	0.137	0.274		−0.47	0.37	0.203	0.406	
		**Daytime ABP levels**												
*SCAP*	rs12487736	male	TT + TC/CC	125/55	3.18	1.53	0.040	0.080	0.048	0.93	0.90	0.303	0.606	0.302
		female		240/90	0.78	1.07	0.468	0.936		−0.30	0.61	0.628	1.000	
	rs76558868	male	GG/AG/AA	60/82/38	1.60	0.95	0.096	0.192	0.064	0.86	0.56	0.122	0.244	0.129
		female		120/169/41	−1.12	0.71	0.118	0.236		−0.50	0.41	0.227	0.454	
		**Nighttime ABP levels**												
*SCAP*	rs12487736	male	TT + TC/CC	125/55	3.62	1.60	0.025	0.050	0.099	2.04	1.02	0.047	0.094	0.160
		female		240/90	0.45	1.03	0.661	1.000		0.06	0.66	0.928	1.000	
	rs76558868	male	GG/AG/AA	60/82/38	2.64	0.98	0.008	0.016	0.027	1.65	0.63	0.009	0.018	0.025
		female		120/169/41	0.77	0.69	0.262	0.524		−0.71	0.44	0.107	0.214	

*SCAP*: sterol regulatory element-binding protein cleavage-activating protein, ABP: ambulatory blood pressure, SNPs: single nucleotide polymorphism, BP: blood pressure, SBP: systolic blood pressure, DBP: diastolic blood pressure, SE: standard error. Adjusted for age, BMI group, ethnicity, monthly household income per capita, salt intake habits, fruit intake frequency, vegetable intake frequency, smoking, drinking, history of hypertension and waist circumference. ^a^: The *p*-values of adjusted multiple testing for Bonferroni correction.

## Data Availability

The datasets analyzed in our study are available from the corresponding author upon reasonable request.

## Supplementary Materials

The following supporting information can be downloaded at: https://www.mdpi.com/article/10.3390/genes17070825/s1, Table S1: Genotype distribution of *SCAP* gene polymorphisms; Table S2: The AIC of different genetic models of *SCAP* gene polymorphisms; Table S3: The association between *SCAP* gene polymorphisms and abnormal ambulatory blood pressure (24-h, daytime, nighttime blood pressure); Table S4: The interaction between *SCAP* gene polymorphisms and weight status on ambulatory blood pressure levels.

## References

[1] Mills K.T., Stefanescu A., He J. (2020). The global epidemiology of hypertension. Nat. Rev. Nephrol..

[2] GBD 2023 Disease and Injury and Risk Factor Collaborators (2025). Burden of 375 diseases and injuries, risk-attributable burden of 88 risk factors, and healthy life expectancy in 204 countries and territories, including 660 subnational locations, 1990–2023: A systematic analysis for the Global Burden of Disease Study 2023. Lancet.

[3] Zhang M., Shi Y., Zhou B., Huang Z., Zhao Z., Li C., Zhang X., Han G., Peng K., Li X. (2023). Prevalence, awareness, treatment, and control of hypertension in China, 2004–2018: Findings from six rounds of a national survey. BMJ.

[4] Zhou J., Shan S., Wu J., Song Y., Zhu L., Li Q., Zhang C., Zhu Y., Sheikh A., Rahimi K. (2025). Global prevalence of hypertension among children and adolescents aged 19 years or younger: An updated systematic review and meta-analysis. Lancet Child. Adolesc. Health.

[5] Omboni S., Bilo G., Saladini F., Di Guardo A., Palatini P., Parati G., Pucci G., Virdis A., Muiesan M.L. (2024). Standards for the Implementation, Analysis, Interpretation, and Reporting of 24-h Ambulatory Blood Pressure Monitoring Recommendations of the Italian Society of Hypertension. High Blood Press. Cardiovasc. Prev..

[6] Evangelou E., Warren H.R., Mosen-Ansorena D., Mifsud B., Pazoki R., Gao H., Ntritsos G., Dimou N., Cabrera C.P., Karaman I. (2018). Genetic analysis of over 1 million people identifies 535 new loci associated with blood pressure traits. Nat. Genet..

[7] Lu Q., Zhang Y., Geng T., Yang K., Guo K., Min X., He M., Guo H., Zhang X., Yang H. (2022). Association of Lifestyle Factors and Antihypertensive Medication Use with Risk of All-Cause and Cause-Specific Mortality Among Adults with Hypertension in China. JAMA Netw. Open.

[8] El Cheikh J., Hamed F., Rifi H., Dakroub A.H., Eid A.H. (2025). Genetic polymorphisms influencing antihypertensive drug responses. Br. J. Pharmacol..

[9] Cheng C., Geng F., Li Z., Zhong Y., Wang H., Cheng X., Zhao Y., Mo X., Horbinski C., Duan W. (2022). Ammonia stimulates SCAP/Insig dissociation and SREBP-1 activation to promote lipogenesis and tumour growth. Nat. Metab..

[10] Yang T., Espenshade P.J., Wright M.E., Yabe D., Gong Y., Aebersold R., Goldstein J.L., Brown M.S. (2002). Crucial step in cholesterol homeostasis: Sterols promote binding of SCAP to INSIG-1, a membrane protein that facilitates retention of SREBPs in ER. Cell.

[11] Sakai J., Nohturfft A., Cheng D., Ho Y.K., Brown M.S., Goldstein J.L. (1997). Identification of complexes between the COOH-terminal domains of sterol regulatory element-binding proteins (SREBPs) and SREBP cleavage-activating protein. J. Biol. Chem..

[12] Esquejo R.M., Roqueta-Rivera M., Shao W., Phelan P.E., Seneviratne U., Am Ende C.W., Hershberger P.M., Machamer C.E., Espenshade P.J., Osborne T.F. (2021). Dipyridamole Inhibits Lipogenic Gene Expression by Retaining SCAP-SREBP in the Endoplasmic Reticulum. Cell Chem. Biol..

[13] Frazier K., Manzoor S., Carroll K., DeLeon O., Miyoshi S., Miyoshi J., St George M., Tan A., Chrisler E.A., Izumo M. (2023). Gut microbes and the liver circadian clock partition glucose and lipid metabolism. J. Clin. Investig..

[14] Meng H., Gonzales N.M., Lonard D.M., Putluri N., Zhu B., Dacso C.C., York B., O’Malley B.W. (2020). XBP1 links the 12-h clock to NAFLD and regulation of membrane fluidity and lipid homeostasis. Nat. Commun..

[15] Douma L.G., Gumz M.L. (2018). Circadian clock-mediated regulation of blood pressure. Free Radic. Biol. Med..

[16] Guan D., Bae H., Zhou D., Chen Y., Jiang C., La C.M., Xiao Y., Zhu K., Hu W., Trinh T.M. (2023). Hepatocyte SREBP signaling mediates clock communication within the liver. J. Clin. Investig..

[17] Yang Y.D., Song J.Y., Wang S., Liu F.H., Zhang Y.N., Shang X.R., Wang H.J., Ma J. (2017). Genetic variations in sterol regulatory element binding protein cleavage-activating protein (SCAP) are associated with blood pressure in overweight/obese Chinese children. PLoS ONE.

[18] Yang Y.D., Song J.Y., Wang S., Wang Y., Song Q.Y., Li C.X., Dong B., Wang H.J., Ma J. (2019). Interaction between lifestyle behaviors and genetic polymorphism in SCAP gene on blood pressure among Chinese children. Pediatr. Res..

[19] Kotsis V., Stabouli S., Bouldin M., Low A., Toumanidis S., Zakopoulos N. (2005). Impact of obesity on 24-h ambulatory blood pressure and hypertension. Hypertension.

[20] Murden R.J., Fields N.D., Martin Z.T., Risk B.B., Alonso A., Manatunga A., Erving C.L., Moore R., Udaipuria S., Quyyumi A. (2025). Associations between obesity class and ambulatory blood pressure curves in African American women. Obesity.

[21] Zhou Y., Zhao L., Zhang Z., Meng X., Cai Q.J., Zhao X.L., Wang L.P., Hu A.H., Zhou X.L. (2024). Sex difference in nocturnal blood pressure dipping in adolescents with varying degrees of adiposity. BMC Pediatr..

[22] Leu H.B., Chung C.M., Lin S.J., Chiang K.M., Yang H.C., Ho H.Y., Ting C.T., Lin T.H., Sheu S.H., Tsai W.C. (2015). Association of circadian genes with diurnal blood pressure changes and non-dipper essential hypertension: A genetic association with young-onset hypertension. Hypertens. Res..

[23] Gao M., Lv J., Yu C., Guo Y., Bian Z., Yang R., Du H., Yang L., Chen Y., Li Z. (2020). Metabolically healthy obesity, transition to unhealthy metabolic status, and vascular disease in Chinese adults: A cohort study. PLoS Med..

[24] Group of China Obesity Task Force (2004). Body mass index reference norm for screening overweight and obesity in Chinese children and adolescents. Chin. J. Epidemiol..

[25] Kidawara Y., Kadoya M., Igeta M., Morimoto A., Miyoshi A., Kakutani-Hatayama M., Kanzaki A., Konishi K., Kusunoki Y., Daimon T. (2024). Nocturnal Hypertension and Left Ventricular Diastolic Dysfunction in Patients with Diabetes with the Absence of Heart Failure: Prospective Cohort HSCAA Study. Hypertension.

[26] Parati G., Stergiou G., O’Brien E., Asmar R., Beilin L., Bilo G., Clement D., de la Sierra A., de Leeuw P., Dolan E. (2014). European Society of Hypertension practice guidelines for ambulatory blood pressure monitoring. J. Hypertens..

[27] (2021). The Chinese Hypertension League (CHL) Guidelines Committee for Home Blood Pressure Monitoring. 2020 Chinese Hypertension League Guidelines on Ambulatory Blood Pressure Monitoring. Chin. Circ. J..

[28] Duan S., Zhang W., Cox N.J., Dolan M.E. (2008). FstSNP-HapMap3: A database of SNPs with high population differentiation for HapMap3. Bioinformation.

[29] Balloux F., Lugon-Moulin N. (2002). The estimation of population differentiation with microsatellite markers. Mol. Ecol..

[30] Yeh J.T., Huang C.J., Lee C.W., Chen Y.J., Huang S.L., Wang W.T., Tu Y.K., Chiu T.J., Chiang C.E., Chen C.H. (2025). Agreement Between Different Types of Blood Pressure Monitoring: A Systematic Review and Network Meta-analysis. Ann. Intern. Med..

[31] Pike M.M., Schildcrout J., Baldwin S., Edwards T., Lipworth L., Robinson-Cohen C. (2023). Genetic Variants Associated with Systolic Blood Pressure in Children and Adolescents. J. Am. Heart Assoc..

[32] Heemers H., Verrijdt G., Organe S., Claessens F., Heyns W., Verhoeven G., Swinnen J.V. (2004). Identification of an androgen response element in intron 8 of the sterol regulatory element-binding protein cleavage-activating protein gene allowing direct regulation by the androgen receptor. J. Biol. Chem..

[33] Shimano H., Sato R. (2017). SREBP-regulated lipid metabolism: Convergent physiology—Divergent pathophysiology. Nat. Rev. Endocrinol..

[34] Kienitz T., Quinkler M. (2008). Testosterone and blood pressure regulation. Kidney Blood Press. Res..

[35] Traish A., Bolanos J., Nair S., Saad F., Morgentaler A. (2018). Do Androgens Modulate the Pathophysiological Pathways of Inflammation? Appraising the Contemporary Evidence. J. Clin. Med..

[36] Guzik T.J., Touyz R.M. (2017). Oxidative Stress, Inflammation, and Vascular Aging in Hypertension. Hypertension.

[37] Meng Y., Zong L. (2019). Estrogen stimulates SREBP2 expression in hepatic cell lines via an estrogen response element in the SREBP2 promoter. Cell. Mol. Biol. Lett..

[38] Colafella K.M.M., Denton K.M. (2018). Sex-specific differences in hypertension and associated cardiovascular disease. Nat. Rev. Nephrol..

[39] Riglea T., Dessy T., Kalubi J., Goulet D., Ikwa Ndol Mbutiwi F., Williams S.M., Engert J.C., Chen H.Y., O’Loughlin J., Sylvestre M.P. (2025). Body mass index modifies genetic susceptibility to high systolic blood pressure in adolescents and young adults: Results from an 18-year longitudinal study. J. Hum. Hypertens..

[40] Nagarajan P., Winkler T.W., Bentley A.R., Miller C.L., Kraja A.T., Schwander K., Lee S., Wang W., Brown M.R., Morrison J.L. (2025). A large-scale genome-wide study of gene-sleep duration interactions for blood pressure in 811,405 individuals from diverse populations. Mol. Psychiatry.

