# Supplementary material for: Association of SCAP Gene Polymorphisms with Ambulatory Blood Pressure Parameters in College Students
Source: Genes (Basel). 2026 Jul 19;17(7):825. doi: 10.3390/genes17070825 (PMC13409555; doi:10.3390/genes17070825)
Supplement: Supplementary file 1 [file genes-17-00825-s001.zip › genes-4330719-supplementary.pdf]

Table S1 Genotype distribution of *SCAP* gene polymorphisms

Genes	SNPs	Length of gene (kb)	Location	Mutation types	Allele		EAF [a]	EAF [b]	F_{ST}	*P* -HWE	Frequency of genotype (11/12/22)		Call rate	Reference
					1	2					Normal ABP	Abnormal ABP [c]		
SCAP	rs12487736	62	47459679	missense mutation	C	T	0.535	0.410	0.016	0.899	134/240/105	11/16/4	100%	[1]
SCAP	rs76558868		47496464	Intron mutation	A	G	0.325	0.158	0.038	0.473	72/237/170	7/14/10	100%	Tag SNP

SCAP: sterol regulatory element-binding protein cleavage-activating protein, SNPs: Single nucleotide polymorphisms, EAF: effect allele frequency, HWE: Hardy-Weinberg equilibrium, ABP: Ambulatory Blood Pressure. Allele: 1 is an effector allele, and 2 is a non-effector allele. a: represents the population of this study, b: represents the European Community. c: The abnormal ABP group refers to the research subjects whose blood pressure is abnormal in at least one of the three abnormal blood pressure indicators: 24-hour blood pressure, daytime blood pressure and nighttime blood pressure. The normal ABP group refers to the research subjects whose three blood pressure indicators are all normal.

Table S2 The AIC of different genetic models of *SCAP* gene polymorphisms

Genes	SNPs	AIC		
		Additive genetic model	Dominant genetic model	Recessive genetic model
SCAP	rs12487736	2114.44	2115.46	**2113.82**
SCAP	rs76558868	**2115.42**	2115.43	2115.55

SCAP: sterol regulatory element-binding protein cleavage-activating protein, AIC：Akaike Information Criterion.

Table S3 The association between *SCAP* gene polymorphisms and abnormal ambulatory blood pressure (24-hour, daytime, nighttime blood pressure)

Genes	SNPs	Genotype	N	Model 1		Model 2	
				***OR* (*95%CI*)**	***P***	***OR* (*95%CI*)**	***P***
**abnormal 24-hour blood pressure**							
SCAP	rs12487736	TT+TC	365	Reference		Reference	
		CC	145	1.51(0.53~4.26)	0.440	1.27(0.39~4.19)	0.694
	rs76558868	GG/AG/AA	79/251/180	1.68(0.82~3.41)	0.155	1.48(0.67~3.28)	0.338
**abnormal daytime blood pressure**							
SCAP	rs12487736	TT+TC	365	Reference		Reference	
		CC	145	1.25(0.43~3.66)	0.680	0.82(0.22~3.13)	0.775
	rs76558868	GG/AG/AA	79/251/180	1.47(0.72~3.02)	0.291	1.19(0.52~2.74)	0.679
**abnormal nighttime blood pressure**							
SCAP	rs12487736	TT+TC	365	Reference		Reference	
		CC	145	1.16(0.44~3.06)	0.759	1.10(0.41~2.96)	0.849
	rs76558868	GG/AG/AA	79/251/180	0.99(0.52~1.89)	0.965	0.92(0.47~1.80)	0.801

SCAP: sterol regulatory element-binding protein cleavage-activating protein, Model 1 adjusted for sex, age and BMI; Model 2 further adjusted for ethnicity, monthly household income per capita, salt intake habits, fruit intake frequency, vegetable intake frequency, smoking, drinking, history of hypertension and waist circumference.

Table S4 The interaction between *SCAP* gene polymorphisms and weight status on ambulatory blood pressure levels

Genes	SNPs	Weight status	Genotype	N	SBP				DBP			
					β	***SE***	***P***	***P interaction***	***β***	***SE***	***P***	***P interaction***
**24-hour Ambulatory Blood Pressure levels**												
SCAP	rs12487736	normal	TT+TC/CC	288/111	1.50	0.91	0.098	0.233	0.83	0.52	0.114	0.077
		overweight/obesity		77/34	-0.52	1.59	0.745		-0.60	0.96	0.532	
	rs76558868	normal	GG/AG/AA	148/191/60	0.42	0.59	0.481	0.533	0.39	0.34	0.251	0.228
		overweight/obesity		32/60/19	-0.44	1.07	0.678		-0.53	0.64	0.411	
**Daytime Ambulatory Blood Pressure levels**												
SCAP	rs12487736	normal	TT+TC/CC	288/111	1.15	1.00	0.253	0.365	0.60	0.58	0.296	0.123
		overweight/obesity		77/34	-0.49	1.86	0.792		-0.83	1.11	0.458	
	rs76558868	normal	GG/AG/AA	148/191/60	0.25	0.65	0.703	0.597	0.33	0.37	0.377	0.135
		overweight/obesity		32/60/19	-0.93	1.24	0.458		-0.74	0.75	0.323	
**Nighttime Ambulatory Blood Pressure levels**												
SCAP	rs12487736	normal	TT+TC/CC	288/111	2.15	0.99	**0.031**	0.153	1.05	0.62	0.088	0.232
		overweight/obesity		77/34	-0.43	1.95	0.825		0.13	1.38	0.924	
	rs76558868	normal	GG/AG/AA	148/191/60	0.59	0.65	0.367	0.228	0.39	0.40	0.334	0.554
		overweight/obesity		32/60/19	0.34	1.31	0.798		-0.18	0.93	0.844	

SBP: Systolic blood pressure, DBP: Diastolic blood pressure. Adjusted for gender, age ethnicity, monthly household income per capita, salt intake habits, fruit intake frequency, vegetable intake frequency, smoking, drinking, history of hypertension and waist circumference.

Reference

1 Yang, Y. D. et al. Interaction between Lifestyle Behaviors and Genetic Polymorphism in Scap Gene on Blood Pressure among Chinese Children. *Pediatr Res* **86**, 389-395 (2019).
